# Repo-Man/protein phosphatase 1 SUMOylation mediates binding to lamin A and serine 22 dephosphorylation

**DOI:** 10.1098/rsob.220017

**Published:** 2022-04-13

**Authors:** Florentin Huguet, Ezgi Gokhan, Helen A. Foster, Hasnat A. Amin, Paola Vagnarelli

**Affiliations:** ^1^ College of Medicine, Health and Life Science, Brunel University London, Centre for Genomic Engineering and Maintenance (CenGem), London UB8 3PH, UK; ^2^ Biosciences, Department of Clinical, Pharmaceutical and Biological Sciences, School of Life and Medical Sciences, University of Hertfordshire, Hatfiled, UK

**Keywords:** protein phosphatase 1, SUMOylation, lamin A, phosphorylation, mitosis

## Abstract

Lamin A phosphorylation/de-phosphorylation is an important process during cells division as it allows for nuclear envelope (NE) disassembly at mitotic entry and its re-assembly during mitotic exit. Several kinases have been identified as responsible for these phosphorylations, but no protein phosphatase has been implicated in their reversal. One of the mitotic phosphosites in lamin A responsible for its dynamic behaviour is serine 22 (S22) which is de-phosphorylated during mitotic exit. Recent evidence has also linked the nuclear pool of lamin A S22ph in interphase to gene expression regulation. Previous work suggested that the phosphatase responsible for lamin A S22 de-phosphorylation is chromatin bound and interacts with lamin A via SUMO-SIM motives. We have previously reported that Repo-Man/protein phosphatase 1 (PP1) is a chromatin-associated phosphatase that regulates NE reformation. Here we propose that Repo-Man/PP1 phosphatase mediates lamin A S22 de-phosphorylation. We indeed show that depletion of Repo-Man leads to NE defects, causes hyperphosphorylation of lamin A S22 that can be rescued by a wild-type but not a SUMOylation-deficient mutant. Lamin A and Repo-Man interact *in vivo* and *in vitro*, and the interaction is mediated by SUMOylation. Moreover, the localization of Repo-Man/PP1 to the chromatin is essential for lamin A S22 de-phosphorylation.

## Graphical abstract

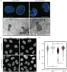

## 1. Introduction

Nuclear lamins are essential component of the nuclear envelope (NE) and exert several functions. They are responsible for the nucleo-skeletal stiffness of cells, an important aspect for both tissue maintenance and metastatic invasion [1,2]. Despite these intermediate filaments providing structure and thus protection to the chromatin within cells, they are also very dynamic. The nuclear size increases from early G1 to S phase, migrating cells reshape their nuclei, and, even more dramatically, dividing cells disassemble the NE as they enter mitosis to rebuild it at mitotic exit.

These dynamic features are mediated by the disassembly and re-assembly of lamins (lamin A and lamin B types). Both the N- and C-terminus of lamin A control the solubility of the protein, and, from a molecular point of view, farnesylation and phosphorylations are the known post-translational modifications that influence lamins dynamics [3]. During mitosis, Cdk1 phosphorylations of residues Serine 22 (S22) and S392 (and similar ones in lamin B) regulate lamin A and B dynamics, although S22 seems more important for lamin head-to-tail de-polymerization, than the C-terminal site [4,5]. Protein kinase C is also involved in phosphorylating lamin B1. Lamin A phosphorylation occurs also in interphase, although it is not known how this regulation occurs. What is known is that lamin A S22ph does not localize at the nuclear periphery, but it is found in the nuclear space and bound to chromatin at a subset of putative active enhancers [6]. Moreover, matrix stiffness coupled to myosin-II activity seems to promote lamin A/C S22 de-phosphorylation to regulate its turnover and physical properties [7].

Despite the importance of lamin structure and dynamics during normal cell cycle and diseases, very little is known on how their post-translational homeostasis is regulated. Several kinases responsible for the phosphorylation of specific sites have been identified, but the counteracting phosphatases are still elusive. The only known phosphatase that counteracts the PKA mitotic phosphosite in lamin B is protein phosphatase 1 (PP1) recruited by the A-kinase anchoring protein AKAP149 [8].

Recently, it was shown that the de-phosphorylation of lamin A S22 is mediated by an interaction between a SUMO interacting motif (SIM) present in the immunoglobulin (Ig) fold of lamin A and an unknown chromosome-associated SUMOylated protein [9].

Here, we show that the chromatin-associated PP1 targeting protein Repo-Man is essential for lamin S22 de-phosphorylation during mitotic exit, thus identifying the first phosphatase responsible for the dynamics regulation of lamin A.

## 2. Material and methods

### 2.1. Cell culture, cloning and transfection

HeLa cells were cultured in a 37°C humid incubator with 5% CO_2_ in DMEM high glucose, GlutaMAX supplement (Gibco) supplemented with 10% FBS (LabTech), 100 U ml^−1^ penicillin and 100 µg ml^−1^ streptomycin (Gibco). Repo-Man-FKBP-FRB cell line was cultured in a 37°C humid incubator with 5% CO_2_ in McCoy's 5A (modified) medium, GlutaMAX supplement (Gibco) supplemented with 10% FBS (LabTech), 100 U ml^−1^ penicillin and 100 µg ml^−1^ streptomycin (Gibco). For plasmid transfection, HeLa cells were seeded in 10 cm dish at 30% confluency 2 days prior transfection. Cells were transfected with 5 µg DNA of GFP alone, GFP-Repo-Man^WT^ or GFP-Repo-Man^IRCE^ constructs for 24 h with JetPRIME (Polyplus Transfection). For siRNA transfection, cells were seeded 24 h prior to transfection at 30% confluency and transfected with 50 nM siRNA (esiRNA-*LMNA*: EHU063791, Merck for lamin A knock down and for Repo-Man and control knock down, previously validated oligos were used [10]) for 48 h with JetPRIME. When needed, cells were treated with 200 ng ml^−1^ nocodazole for 16 h.

A CRISPR/Cas9 plasmid was constructed from pX330-U6-Chimeric_BB-CBh-hSpCas9 (no. 42230, Addgene). The guide RNA were designed with CRISPOR (http://crispor.tefor.net), and inserted into the CRISPR/Cas9 plasmid following Ran *et al*. protocol [11]. To construct donor plasmids, genomic DNA from HCT116 (gift from Prof. Kanemaki, Japan) was purified by using EchoLUTION CellCulture DNA Kit (BioEcho), and 1200 bp genomic DNA region around the *CDCA2* stop codon was amplified using Phusion High-Fidelity DNA Polymerase (Thermo Fisher Scientific) (forward primer: 5′-AGACGTTCCATGTGTTATTCTGATG-3′, reverse primer: 5′-CATATCTTACCCGCTTTAAGTCTTC-3′), cloned in pGEM-T Easy empty vector (Promega) and sequenced. Based on sequencing, the plasmid containing *CDCA2* homology arms was synthetized with PAM point mutations in PAM sequences to avoid the re-cutting by Cas9, and the *CDCA2* stop codon was mutated to generate a BamHI restriction site. FKBP was obtained by PCR from pLEX-FKBP-Rab11-Blasticidin (no. 120717, Addgene) and inserted in pMK-289 (no. 72827, Addgene) and pMK-290 (no. 72831, Addgene) using SacI/NheI restriction sites, thus replacing mAID by FKBP. pMK-289 and pMK-290 containing FKBP were inserted between *CDCA2* homology arms at the BamHI site. pMito-mCherry-FRB was obtained from Addgene (no. 59352, Addgene).

### 2.2. Site-directed mutagenesis

All mutations were performed by using GENEART Site-Directed Mutagenesis System and AccuPrimeTM Pfx DNA Polymerase (Invitrogen). The DNA concentration to be used was optimized to 100 ng µl^−1^ and a PCR mixture was prepared for each reaction by using specifically designed oligonucleotides (Eurofins Genomics, Germany).

### 2.3. Immunofluorescence and microscopy

Cells were fixed with 4% PFA for 5 min and permeabilized with 0.2% Triton X-100 for 2 min and then blocked in 1% BSA for 30 min at 37°C. Antibody dilutions were prepared in 1% BSA. Slides were counterstained with DAPI.

Three-dimensional datasets were acquired using a wide-field microscope (NIKON Ti-E super research Live Cell imaging system) with a NA 1.45 Plan Apochromat lens. The datasets were deconvolved with NIS Elements AR analysis software (NIKON). Three-dimensional datasets were converted to Maximum Projection in the NIS software, exported as TIFF files and imported into Adobe Photoshop for final presentation.

For the quantification of lamin A/C-S22ph, three-dimensional stack images were acquired with the same exposure. Using NIS-Element AR, an area corresponding to chromosomes or the nuclei was used to measure the mean intensity of lamina S22ph signals. For the total lamin A/C-S22ph, an area containing all lamin A/C S22ph signals was selected and used to measure mean intensity of total lamin A/C-S22ph. Areas of the same size within each image were used to identify and subtract the background from the measurements.

For the quantification of the nuclear circularity, the NIS Elements AR analysis software (NIKON) was used.

Violin plots were generated using the ggplot2 package in R.

### 2.4. Electron microscopy

HeLa cells (1 × 10^5^) were seeded into µ-dish 35 mm imaging dishes containing a coverslip at the bottom with a cell location grid (Ibidi). HeLa cells were transfected with 400 ng lamin GFP and 20 mM of either control siRNA or Repo-Man siRNA using jetPRIME transfection reagent (Polyplus transfection) for 48 h as recommended in the manufacturer's instructions. Live cell images and grid coordinates were captured at 37°C in 5% CO_2_ using a 63× objective on a NIKON Ti-E super research Live Cell imaging system microscope and NIS-Element software.

Cells within the imaging dishes were fixed with 3% glutaraldehyde (Agar Scientific) and 0.5% PFA (Agar Scientific) in 0.05 M phosphate buffer (PB; 0.05 M was prepared by mixing 101.25 ml 0.2 M Na_2_HPO_4_ pH 7.4 with 23.75 ml of 0.2 M NaH_2_PO_4_.2H_2_O pH 7.4, to a final volume of 500 ml with water) for 1 h at room temperature. The fixative was removed prior to two 15-min rinses in wash solution (0.05 PB, 0.1 M sucrose) and one 15-min wash with distilled water. Cells were incubated with 1% osmium tetroxide (Agar Scientific) for 1 h at room temperature and subsequently washed twice in distilled water for 15 min and then 30 min in 30% ethanol. The ethanol was decanted and replaced with 0.5% uranyl acetate (TAAB) in 30% ethanol for a 1 h incubation before being decanted and going through an ethanol series of 30%, 50%, 60%, 70%, 80, 90% ethanol and twice in 100% ethanol with a 10-min incubation for each step. Resin was prepared by mixing the following reagents: 20 ml Agar 100 epoxy resin (Agar Scientific), 16 ml dodecenyl succinic anhydride hardener (Agar Scientific), 8 ml methyl nadic anhydride hardener (MNA; Agar Scientific) and 1.3 ml benzyl dimethylamine accelerator (Agar Scientific); note the resin and hardeners were preheated to 60°C before mixing. Cells were incubated with a 1 : 2 ratio of resin: ethanol solution for 20 min, 1 : 1 ratio of resin: ethanol solution for 20 min and finally the resin: ethanol mix was removed and 100% resin was added to the dish and allowed to set for 48 h at 60°C. The coverslip and 35 mm dish were removed, and the cell grid coordinates etched on the resin were identified to determine the cell areas of interest. Excess resin was removed and trimmed, so the cell areas of interest could be sectioned using a PowerTome XL Ultramicrotome. Sections of 100 nm were collected on G200 Hex copper 3.05 mm grids (TAAB) that were formvar coated. Cell sections on the copper grids were post-stained for 7 min in 5% uranyl acetate (TAAB) made in 50% ethanol, rinsed for 1 min in distilled water and incubated in lead citrate for 7 min. To prepare lead citrate, 30 µl 1 M lead nitrate (Analar R) was mixed with 30 µl 1.5 M tri-sodium citrate (TAAB) and 160 µl water and was incubated for 30 min prior to the addition of 40 µl 1 M NaOH (BDH) and then was filtered using a 0.45 µM filter to remove and residual precipitate; note all solutions were prepared in boiled water to ensure any dissolved CO_2_ was removed. The grids were rinsed in distilled water for 1 min and allowed to air dry on filter paper for at least 2 h prior to microscopy. Images were captured on a JEM-2100F field emission electron microscope (JEOL) at 80 kV in the Experimental Technique Centre at Brunel University London.

### 2.5. Proximity ligation assay

Duolink (Sigma, Germany) was used for the proximity ligation assay (PLA). Cells were grown, fixed and blocked on coverslips and primary antibodies (anti-GFP (mouse) (cat no. 11814460001, ROCHE), and anti-lamin A/C S22ph (rabbit) (cat no. 13448, Cell Signaling Technology)) were applied as described in the immunofluorescence section. PLA probes (provided with the kit) were diluted 1 : 5. Samples were incubated for 1 h at 37°C and washed with 1× wash buffer A (provided with the kit) for 5 min (two times). The ligation stock (1 : 5) and ligase (1 : 40) mix were diluted as instructed and applied on the coverslips. After 1 h incubation at 37°C, 1× wash buffer A was applied for 2 min (two times). Amplification stock (1 : 5) and the polymerase (1 : 80) were diluted as instructed and applied on coverslips. After a 100 min incubation at 37°C, samples were washed in 1× wash buffer B for 10 min (two times) and 0.01× wash buffer B for 1 min. Mounting medium (Vectashield) with DAPI was applied, and samples were prepared for imaging.

PLA signals were counted using object detection by thresholding (ROI) using the NIKON software.

### 2.6. Repo-Man-FKBP-FRB cell line generation

Repo-Man-FKBP-FRB cell line was generated from HCT116 expressing TET-OsTIR1. Cells were seeded in six-well plate 2 days prior to transfection. Cells were transfected with the CRISPR/Cas9 plasmid and the two donor plasmids at equal ratio by using JetPRIME. After 48 h, cells were diluted and transferred in 10 cm dishes. Cells were grown for 15 days with media containing 700 µg ml^−1^ G418 (Gibco) and 100 µg ml^−1^ hygromycin B (Thermo Fisher Scientific). The medium was changed every 4 days. Colonies were amplified and FKBP integration was checked by PCR on genomic DNA:

primer F: 5′-GCATCAGATAGTCCCAAACC-3′,
primer R: 5′-TCCCCCCAACACACAACAAA-3′,
primer N: 5′-CGTTGGCTACCCGTGATATT-3′,
primer H: 5′-GCTGTGTAGAAGTACTCGCC-3′.

Once the Repo-Man-FKBP cell line was validated, cells were seeded in six-well plate and co-transfected for 48 h with the pMito-mCherry-FRB plasmid and a second plasmid containing the blasticidin resistance cassette at a 10 : 1 ratio. Cells were diluted and transferred in 10 cm dishes and grown for 15 days with media containing 700 µg ml^−1^ G418, 100 µg ml^−1^ hygromycin B and 10 µg ml^−1^ blasticidin (GIBCO). The medium was changed every 4 days. To confirm the efficiency of the FKBP-FRB system and Repo-Man re-localization, cells were seeded on coverslips. After 3 days, cells were treated with 100 nM rapamycin (Merck), incubated 10 min at 37°C and inspected by microscopy.

### 2.7. Genomic DNA extraction

Genomic DNA extraction was conducted by resuspending the cells in lysis buffer (100 mM Tris-HCl, pH 8.0, 200 mM NaCl, 5 mM EDTA, 1% SDS and 0.6 mg ml^−1^ proteinase K) for 2 h at 55°C in. After centrifugation (10 000*g* × 10 min × 4°C), equal volume of isopropanol was added to the supernatant, and the precipitated DNA was pelleted by centrifugation (20 000*g* × 30 min × 4°C). DNA pellets were washed twice with 70% ethanol, dried and resuspended by incubation overnight at 37°C in water containing 50 µg ml^−1^ RNase A.

### 2.8. GFP immunoprecipitation

Transfected HeLa cells were lysed and sonicated in 600 µl of lysis buffer (10 mM Tris-HCl, pH 7.5, 150 mM NaCl, 0.5 mM EDTA, 0.5% IGEPAL CA-630, 20 mM N-ethylmaleimide (NEM)) in presence of proteases and phosphatases inhibitors cocktail. After centrifugation (20 000*g* × 30 min × 4°C), the supernatant was incubated overnight at 4°C with 50 µl GFP-TRAP magnetic agarose beads (Chromotek) then washed three times with 500 µl washing buffer (10 mM Tris-HCl, pH 7.5, 150 mM NaCl, 0.5 mM EDTA, 0.05% IGEPAL CA-630, 20 mM NEM) in presence of proteases and phosphatases inhibitors cocktail.

### 2.9. Lamin A expression and purification

His-laminA (gift from Dr Makarov, Brunel University London) was expressed in *E. coli* Rosetta. Fifty millilitres of bacteria culture was pelleted. The pellet was resuspended and sonicated in 7 ml lysis buffer (20 mM HEPES pH 8.0, 100 mM NaCl, 8 M Urea, 10 mM imidazole) for 2 h at 4°C. The lysate was centrifuged (16 000*g* × 15 min × 4°C), and His-lamin A was purified from supernatant on TALON beads (Merck) following the manufacturer's instructions. His-lamin A was eluted from TALON beads with 500 µl of lysis buffer containing 50 mM imidazole. The expression and purification of His-lamin A was analysed by SDS-PAGE and Coomassie staining.

### 2.10. *In vitro* binding assay

Two hundred microlitres of PBS and 30 µl (of 500 µl elution) of purified lamin A were mixed with 300 µl beads of GFP alone, GFP-Repo-Man^WT^ or GFP-Repo-Man^IRCE^. The mix was incubated for 2 h at 4°C, washed three times with cold PBS and boiled in 2× Laemmli buffer.

### 2.11. Western blotting

Cells were pelleted and prepared for blotting through sonication in SDS sample buffer. Membranes were incubated with primary antibodies as in and subsequently with IRDye-labelled (Rabbit680RD, 926-68071, LI-COR; Mouse800CW, 926-32210, LI-COR) or HRP-conjugated secondary antibodies (RabbitHRP, 31460, Abcam MouseHRP, 31444, Abcam). Fluorescence intensities were determined using a LI-COR Odyssey CCD scanner according to manufacturer's instructions (LI-COR Biosciences). Anti-SUMO antibody was no. 4971 (Cell Signaling Technology).

### 2.12. Quantification lamina/C S22ph

Quantification of lamin A/C S22ph (total and on chromosomes) was conducted from images of late anaphases of untreated cells and cells treated with 100 nM rapamycin for 5 h. Images were acquired from the same slide and with the same exposure. Using NIS-Element AR, an area corresponding to chromosomes was used to measure mean intensity of lamin A/C S22ph signal. For the total lamin A/C S22ph, an area containing all lamin A/C S22ph signal was selected and used to measure mean intensity of total lamin A/C S22ph. Areas of the same size were used to identify and subtract the background from measurement.

### 2.13. Bioinformatics

The Repo-Man dataset was extracted from the publicly available data (accession no. GSE84035) as described in de Castro *et al*. (2017) [12].

The lamin A data were generated by Lund *et al.* [13] and are also publicly available (GEO accession no. GSE57149). The percentage overlap was calculated by finding the total length of all regions occupied by both proteins (Repo-Man and lamin AC/lamin AC S22) and dividing by the total length of regions occupied by the protein of interest (Repo-Man). To calculate the *p*-value, we simulated Repo-Man datasets as described in de Castro *et al*. [14] and calculated the proportion of times a simulated Repo-Man dataset had a greater percentage overlap than the original.

## 3. Results

### 3.1. Repo-Man/PP1 regulates nuclear morphology

**Figure 1.**
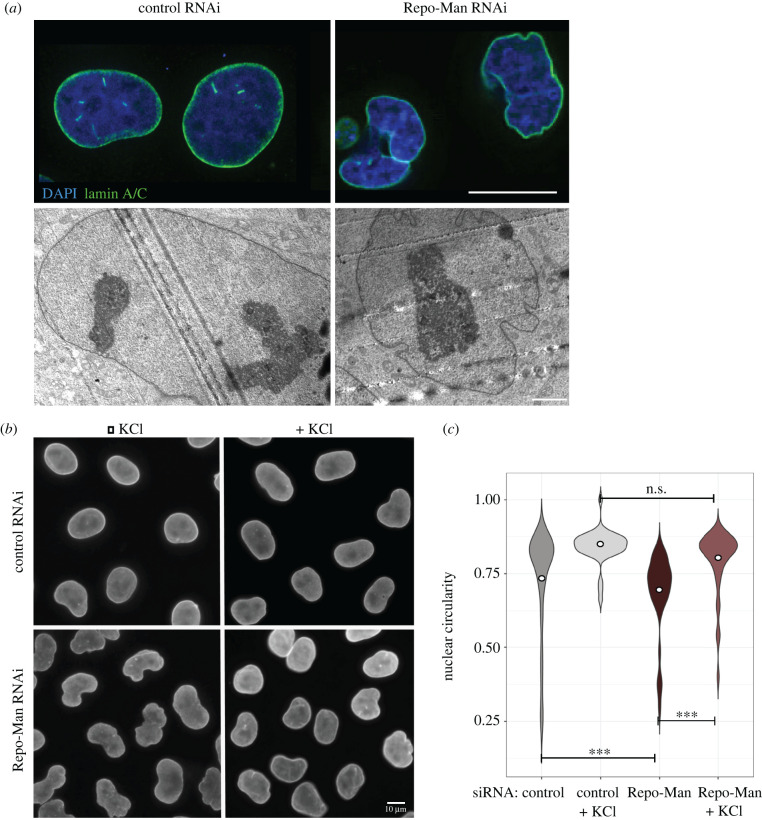
Repo-Man depletion compromises nuclear morphology. (*a*) HeLa cells were treated with control or Repo-Man siRNA oligos. Cells were either fixed and stained with lamin A/C antibodies (green) (top panels) or processed for EM (bottom panel). Scale bar, 15 µm. (*b*) HeLa cells were treated with control or Repo-Man siRNA oligos and fixed either before or after treatment with hypotonic solution (KCl) for 15 min. (*c*) Violin plots representing the analyses of nuclear circularity form images of the experiment in (*b*). At least 200 cells were analysed. The dot represents the median of the distributions. The data were statistically analysed with a Wilcoxon test. n.s., not significant; ***, *p* < 0.001.

We have previously identified the PP1 targeting subunit Repo-Man as an important regulator of chromatin reorganization and NE reformation during mitotic exit. Depletion of Repo-Man leads to abnormally deformed nuclei with defects in both nuclear pore complex and heterochromatin [10,12]. We wanted to investigate in more detail the nuclear morphology defects (figure 1*a*, top panels). To this purpose, we used electron microscopy to look at the nuclear membrane morphology upon control and Repo-Man RNAi. The analyses indicated that the NE defects observed by immunofluorescence were due to the presence of several invaginations (figure 1*a*, bottom panels). This phenotype could be suggestive of either a weaker NE or structural defects in its assembly. To probe this aspect, we subjected cells to hypotonic treatment; in case of major structural defects, it was expected that the nucleus would increases in size but the defects persist. However, if the underlying problem was caused by a weak NE, the treatment with a hypotonic solution would both expand the nucleus and rescue the smooth circularity of the nuclei. We therefore analysed the nuclear circularity of HeLa cells before and after hypotonic treatment in both control and Repo-Man RNAi. These analyses revealed that Repo-Man RNA indeed caused reproducible defects in nuclear circularity, but these abnormalities were rescued by hypotonic treatments (figure 1*b,c*).

### 3.2. Repo-Man depletion leads to increased lamin A S22 phosphorylation

**Figure 2.**
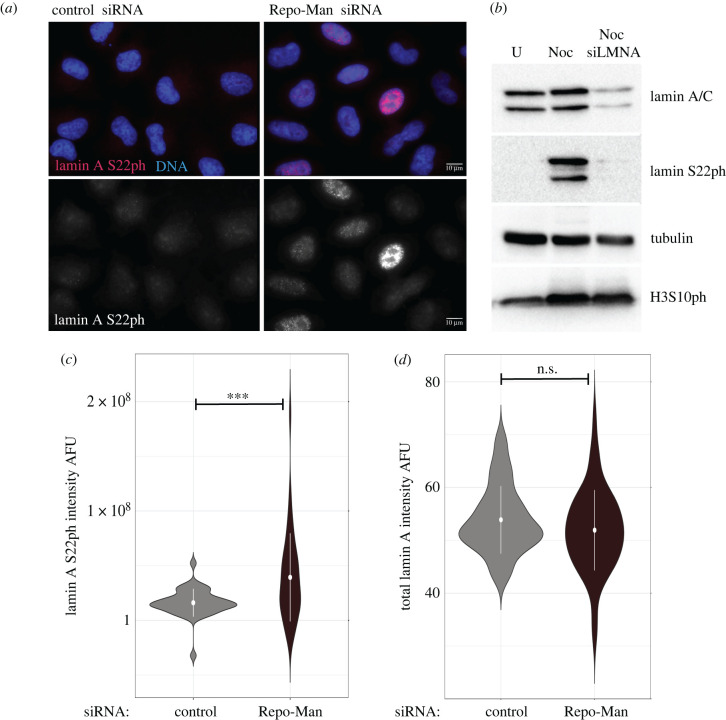
Repo-Man depletion affects lamin A S22 de-phosphorylation. (*a*) HeLa cells were treated with control or Repo-Man siRNA oligos. Cells were fixed and stained for lamin A S22ph (red). (*b*) Western blot of HeLa cells treated with either Control or *LMNA* si oligos in the absence or presence of nocodazole (Noc). Whole-cell lysates were blotted with antibodies against lamin A/C (top panel), lamin A S22ph (second panel), tubulin (third panel) and H3S10ph (bottom panel). (*c*) Violin plots representing the quantification of lamin A S22 intensity on nuclei from the experiment in (*a*). At least 200 cells were analysed. The dot represents the median of the distributions. The data were statistically analysed with a Wilcoxon test. ***, *p* < 0.001. (*d*) Violin plots representing the quantification of total lamin A/C intensity on nuclei of HeLa cells treated with control or Repo-Man siRNA. At least 200 cells were analysed. The dot represents the median of the distributions. The data were statistically analysed with a Wilcoxon test. n.s., not significant.

A weak NE could be caused by several factors including chromatin perturbations such as decreased heterochromatin, changes in lamin A levels or presence of progerin [15]. It is also known that GFP:lamin A S22D mutant is dispersed in the nuclear space and is more mobile [16]; in this respect, increasing the ratio of phospho/non-phospho lamin A would reduce the pool of lamin A available for polymerization and assembly at the NE with the potential of weakening the structure. We have already shown that Repo-Man depletion causes defects in heterochromatin in interphase [12] and we also know that this phosphatase complex acts at the nuclear periphery (directly binds to importin *β* and Nup153). Since lamin A is de-phosphorylated during mitotic exit and accumulates on the chromatin in telophase (electronic supplementary material, figure S1A), we thought to investigate if the level of lamin A S22 phosphorylation was affected upon Repo-Man RNAi. We first verified that the commercial antibody against lamin A/C S22ph was indeed specific for lamin A (as lamin B has the same residue). Western blotting analyses of cells treated with nocodazole (to block cells in prometaphase and to increase the levels of S22 phosphorylation) showed no signal upon *LMNA* RNAi thus indicating that the antibody could be considered specific to perform the subsequent analyses (figure 2*a*). Using this antibody, we then stained cells treated with control or Repo-Man siRNA oligos and quantified the level of lamin A S22ph in interphase. These experiments clearly showed a significant increase in lamin A S22ph in Repo-Man depleted cells (figure 2*b*,*c*); however, the total lamin A levels were unchanged (figure 2*d*).

### 3.3. Repo-Man SUMOylation is necessary for lamin A S22 de-phosphorylation

**Figure 3.**
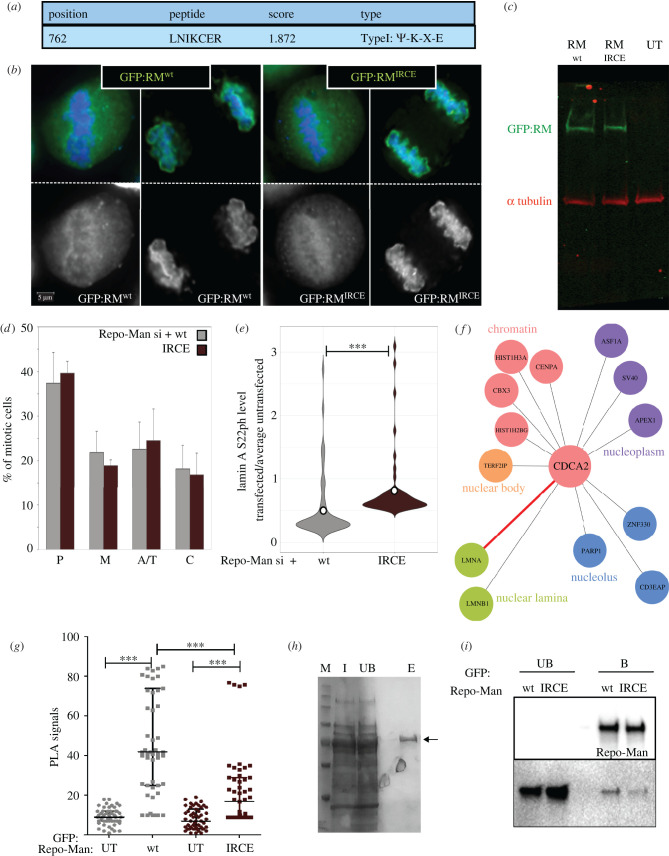
Repo-Man SUMOylation is important for lamin A S22 de-phosphorylation. (*a*) GPSSUMO analyses report for Repo-Man indicating a SUMOylation site at position (aa) 762. (*b*) Representative images of HeLa cells transfected with either GFP:Repo-Man^wt^ (left panels) or GFP:Repo-Man^IRCE^ mutant (right panels) in cells treated with Repo-Man siRNA. (*c*) Western blot of HeLa cells untransfected or transfected with either GFP:Repo-Man^wt^ or GFP:Repo-Man^IRCE^ mutant probed with anti-GFP antibody (green) or alpha tubulin antibody (red). (*d*) HeLa cells were treated with control siRNA or Repo-Man oligos and rescued with ether the oligo-resistant Repo-Man wt or its IRCE mutant. Cells were fixed 48 h post transfection, and the mitotic progression was analysed. P (prometaphase), M (metaphase), A/T (anaphase/telophase) C (cytokinesis). The data represents the average of three biological replicates and 300 cells were analysed per each condition. The error bars indicate the SD. The distribution was statistically analysed by χ^2^-test and the difference between the two distribution was non statistically significant. (*e*) HeLa cells were treated with Repo-Man siRNA oligos, transfected with either GFP:Repo-Man^wt^ or GFP:Repo-Man^IRCE^ mutant for 36 h then fixed and stained for anti-lamin AS22ph. The intensity of nuclear lamin AS22ph staining was calculated for both GFP negative (untransfected) and GFP positive (transfected cells). The violin plot represents the distribution of the ratios between transfected and the average of untransfected cells for each experiment. (*f*) Interaction diagram based on the data obtained by Bio-ID with lamin A [17]. (*g*) Quantification of PLA signals obtained in HeLa cells transfected with either GFP:Repo-Man^wt^ or GFP:Repo-Man^IRCE^ mutant. The primary antibodies were anti-GFP and anti-lamin A S22. (*h*) Lamin A was expressed in *E. coli* and purified according to the material and methods provided. Coomassie staining of the samples for the input (*i*), the unbound fraction (UB) and the elution fraction (E). M = molecular marker. (*i*) HeLa cells were transfected with either GFP:Repo-Man^wt^ or GFP:Repo-Man^IRCE^ mutant. The GFP constructs were pull-down with GFP-binder beads and incubated with recombinant purified lamin A (from *g*). Western blot of the unbound (UB) and bound (B) fractions probed with anti-GFP (top panel) or lamin A (bottom panel) antibodies.

The phosphatase/s responsible for lamin A de-phosphorylation are not known. The mitotic phosphorylation of the S22 residue is removed during anaphase/telophase, and recent work has shown that it is dependent on a SIM present in the Ig fold domain of lamin A [9]. Analyses of the Repo-Man sequence with GPS-SUMO (http://SUMOsp.biocuckoo.org) revealed a putative SUMOylation site at the position 762 (figure 3*a*). Moreover, a proteomic study has identified Repo-Man as a SUMO2/3 target in HeLa cells [17]. The function of this putative SUMO site in Repo-Man has never been investigated. We therefore generated a Repo-Man mutant where the motif IKCE was changed to IRCE (electronic supplementary material, figure S2A). This construct localizes as the wild-type in HeLa cells during mitosis (figure 3*b*), expresses at similar levels (figure 3*c*) and does not alter mitotic progression (figure 3*d*). GFP:Repo-Man pulled down from HeLa cells is recognized by a SUMO2/3 antibody, and the IRCE mutant shows a decreased signal (electronic supplementary material, figure S2B and D).

These constructs were made oligo-resistant and used in rescue experiments to assess the level of lamin A S22ph phosphorylation upon Repo-Man RNAi. While the wt construct was efficient in reducing the phosphorylation levels compared to the untransfected cells, the IRCE mutant still maintained a higher level of S22ph (figure 3*e*; electronic supplementary material, figure S1B), thus suggesting that this residue was important for lamin A S22 de-phosphorylation. Our results indicate that Repo-Man could indeed be the protein that binds to lamin A SIM3 motif to mediate the phosphosite de-phosphorylation.

The analyses of several published proteomic studies [10,18,19] did not show lamin A in the list of Repo-Man interacting proteins; however, we should note that phosphatase substrates can be very transient interactors and possibly not captured by conventional pull-down experiments. Approaches such as Bio-ID or APEX have more the potential for identifying such interactors. In fact, a very recent study using Bio-ID with lamin A as bait was able to identify Repo-Man as prey [20], suggesting that the interaction between the proteins could be a possibility (figure 3*f*).

To test this, we performed a PLA with anti-GFP and lamin A S22ph antibodies. The analyses of the PLA signals confirmed that the IRCE mutation decreased the interactions between Repo-Man and lamin A (to be noted that the overall levels of lamin A S22 are higher for the IRCE mutants but, nevertheless there are less PLA signals) (figure 3*g*; electronic supplementary material, figure S1C). However, a PLA signal could also be the result of the ability of assembling a multi-protein complex, and it is not a demonstration of direct interaction but rather of close proximity within the nuclear space. To further confirm these findings, we tested if lamin A/C was able to interact with Repo-Man *in vitro*. To this purpose, we pulled down in the presence of NEM (an inhibitor of desumoylase enzymes) GFP:Repo-Man^wt^ or GFP:Repo-Man^IRCE^ from HeLa cells transfected with the indicated constructs, and we conducted *in vitro* binding experiments with recombinant human lamin A protein expressed in *E. coli* (figure 3*h*). Our results indicate that the mutation in the SUMOylation site of Repo-Man decreases the binding to lamin A (figure 3*i* and electronic supplementary material, figure S2C). Taken altogether, these data suggest that that Repo-Man interacts with lamin A, the interaction is enhanced by the SUMOylation of residue K762 in Repo-Man and this interaction promotes the de-phosphorylation of S22 in lamin A.

### 3.4. Repo-Man recruitment to the chromatin is necessary for lamin A S22 de-phosphorylation

**Figure 4.**
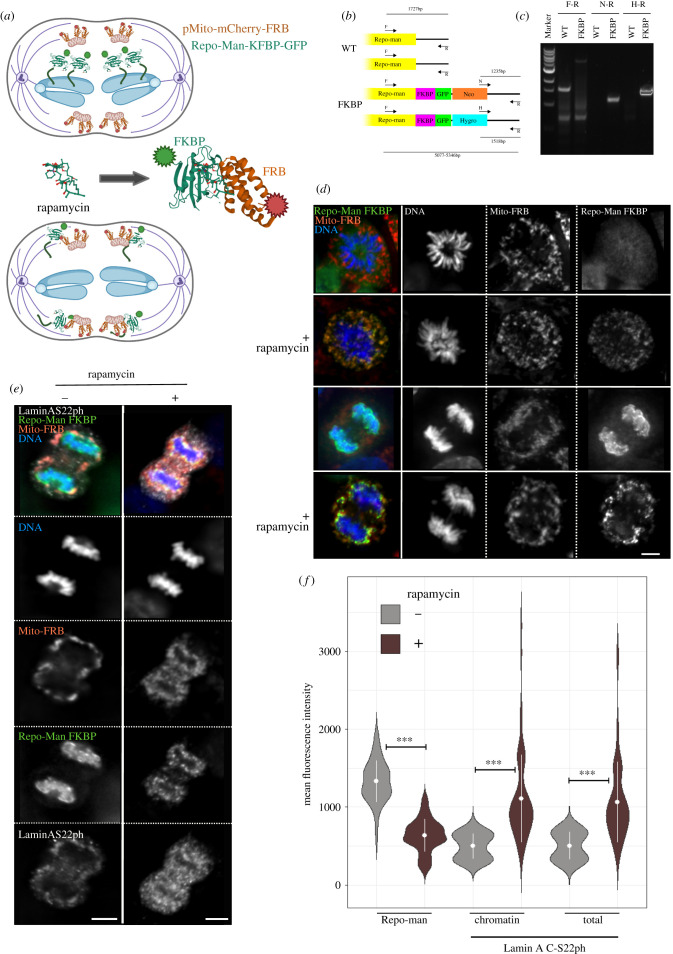
Repo-Man localization on chromatin at anaphase is necessary for lamin A S22 de-phosphorylation. (*a*) Schematic of the cell line and experiment. Repo-Man is endogenously tagged with GFP and FKBP. The cell line also expressed pMito-mCherry-FRB. In anaphase, upon addition of rapamycin FKBP will bind FRB, this removes Repo-Man from the chromatin. Image prepared with Biorender. The crystal structures are from PDB 5GPG [21]. (*b*) Generation of the cell line HCT116 expressing TET-OsTIR1. Both endogenous alleles were tagged with FKBP:GFP. F and R indicate the forward and reverse primers positions respectively. H and N indicate the primers for the hygromycin (H) and neomycin (N) cassettes. The numbers indicated the expected sizes for both the wt and targeted alleles. (*c*) Gel electrophoresis of the PCR were conducted on genomic DNA of the wt cells (WT) or the double targeted cell line (FKBP). Marker is N3232, New England Biolabs. (*d*) Images from prometaphases (top panels) or anaphases (bottom panels) of the HCT116 Repo-Man:FKBP:GFP (green)/pMito-mCherry-FRB (red) with and without rapamycin. Scale bar, 5 μm. (*e*) Representative images of anaphase cells HCT116 Repo-Man:FKBP:GFP (green)/pMito-mCherry-FRB (red) with and without rapamycin and stained for lamin A S22ph (white). (*f*) Violin plots of the quantification of Repo-Man levels and lamin A S22ph on cells from the experiment in (*e*). Statistical analyses were conducted using Wilcoxon test.

Repo-Man is recruited stably to the chromatin at anaphase onset, but it also has some important functions in early mitosis. Since we have so far used an RNAi approach, we wanted to rule out that the effect on anaphase was not caused by defects that occurred earlier in the cell cycle. Moreover, we wondered whether the localization to the chromatin represents an essential part of the process. To address these aspects, we generated a cell line where the endogenous Repo-Man alleles were tagged with FKBP fused to GFP (figure 4*a*,*b*). The cell line is also stably expressing FRB fused to mCherry and a mitochondria localization peptide (pMito-mCherry-FRB). Upon addition of rapamycin, FKBP dimerizes with FRB, thus displacing Repo-Man from the chromatin and relocalizing the complex to the mitochondria (figure 4*a*,*d*). This knock sideway technique has been widely used for structural proteins but never for phosphatase complexes. Using this approach, we can remove the Repo-Man/PP1 complex from the chromatin, but the complex is still active; therefore, potentially, it could still de-phosphorylate proteins that reach the complex by diffusion. Using this tool, cells were arrested in metaphase with MG132 and then released in anaphase by MG132 wash-out in the presence or absence of rapamycin and stained for lamin A S22ph. The removal of Repo-Man from the chromatin does not alter the stages of mitotic progression (electronic supplementary material, figure S2D); therefore, this system can be used for these analyses. The level of Repo-Man on chromatin was quantified together with the total or chromatin-associated lamin A S22ph levels (figure 4*e*,*f*). As the data show, rapamycin depleted Repo-Man from chromatin efficiently, and this led to an increase of lamin A S22ph in both compartments. The data suggest that lamin A S22 de-phosphorylation is indeed a process that depends on Repo-Man/PP1 complex in anaphase and that Repo-Man localization to the chromatin is essential for this process. One possible explanation is that the ubiquitin ligase that targets Repo-Man is chromatin associated (there are lots of SUMOylated protein associated with chromosomes); therefore, lack of Repo-Man targeting would decrease the affinity of the complex for lamin A.

Interestingly, we did not see co-localization of Repo-Man and lamin AS22ph when Repo-Man is delocalized to the mitochondria, suggesting that maybe other factors are involved in the recruitment of lamin A to the chromatin and then the SUMOylation of Repo-Man is important for stabilizing the binding and mediating its de-phosphorylation.

We have also investigated if a Repo-Man mutant that was described not to bind chromatin (S893D) [22] was able to be SUMOylated. Our results suggested that this is the case (electronic supplementary material, figure S3A). However, we have also observed that although this mutant does not accumulate on the chromatin as the wt, it still binds to chromatin weakly (electronic supplementary material, figure S3B); this observation could suggest that even a short binding time is sufficient for the modification to occur.

**Figure 5.**
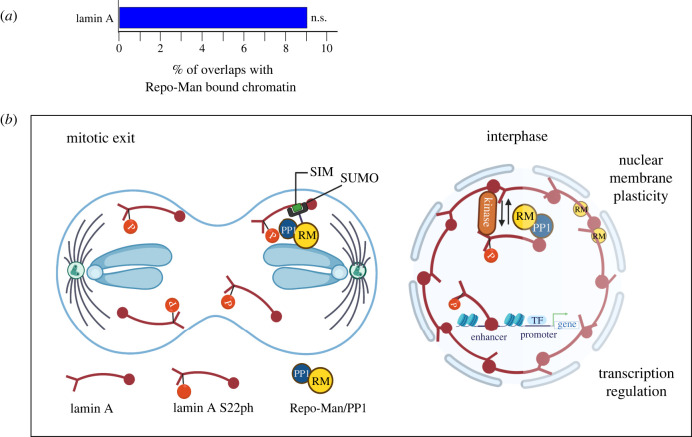
Repo-Man mediates the de-phosphorylation of lamin A S22 but does not with lamin A. (*a*) Percentage overlap between Repo-Man bound chromatin and lamin A (blue). n.s., not significant (see §2). (*b*) Model for Repo-Man/lamin A interaction at mitotic exit (left) and a possible role in interphase (right).

These findings have also wider implications. In fact, lamin A S22 is also phosphorylated in interphase, albeit at lower levels, and associates with chromatin [6]. Repo-Man has a role in interphase as well: it is present both at the nuclear interior and associated to chromatin. Approximately 20% of Repo-Man is indeed chromatin bound as revealed by cellular fractionation [12] and FRET analyses [23]. We then wondered whether chromatin bound Repo-Man was maybe co-localizing with the lamina associated chromatin. Repo-Man is enriched at the nuclear periphery [12], but our PLA data did not show enrichment in this compartment; on the contrary, the signals were in the nuclear interior. Lamin A S22 is also located in the nuclear interior in interphase and a fraction is bound to chromatin at enhancer where it regulates the transcription of several genes. The rest is in a dynamic exchange: the phosphorylated (S22ph)-lamin A is not proficient in assembling into filaments and is released from the NE. Therefore, the balance between phosphorylated and non-phosphorylated lamin A plays a role in regulating the NE rigidity. Repo-Man could therefore be important in these dynamics. We therefore conducted a bioinformatic analysis to assess the overlaps between Repo-Man bound chromatin and lamin A bound chromatin. The datasets were obtained from HeLa cells and previously published [12,13]. We could not detect any significant overlaps between Repo-Man and lamin A (figure 5*a*). We therefore conclude that the interactions between Repo-Man and lamin A are more likely to occur in the soluble fraction of the nucleoplasm thus suggesting a role for Repo-Man more towards controlling nuclear rigidity (figure 5*b*).

## 4. Discussion

Here, we have provided evidence that strongly supports the involvement of Repo-Man/PP1 phosphatase in the de-phosphorylation of the S22 site on lamin A.

While this manuscript was under revision, a similar article was published showing that Repo-Man is indeed SUMOylated at the residue R762 and that a mutation at this site compromises lamin A S22 de-phosphorylation [24]. In this respect, our two independent investigations show exactly the same results [24] have reported that Repo-Man SUMOylation seems to increase during late mitosis (in telophase); however, we do also see some PLA signals in interphase (electronic supplementary material, figure S1C). This could indicate that a fraction of Repo-Man could also be SUMOylated transiently outside mitosis when the NE is in need of reorganization. Future studies will evaluate when this occurs.

Lamin A is an important structural component of the NE; it is expressed in most differentiated cells at low levels, as well as in embryonic stem cells and in the inner cell mass of blastocysts [25].

Several mutations within the *LMNA* gene have been associated with diseases that are collectively referred to as laminopathies [26]. Lamin A has a major contribution towards the regulation of the nuclear shape and stiffness but also participates in the modulation of chromatin organization, DNA replication and transcription. Changes in nuclear shape and stiffness are an integral part of cells. For example, during mitosis the NE is disassembled as cells enter mitosis, and it is reassembled during mitotic exit; in addition, cells that undergo migration (an important process in embryogenesis, wound healing, immune responses and progression of metastatic cancers) need to modify the nuclear structure and stiffness to allow for deformation but preventing rupture.

Xenograft studies have shown that lamin A levels can affect tumor growth. High levels of lamin A in cancers [27] could act as pro-survival feature in stressful migration of the metastatic cells thus protecting from post-migration apoptosis [28].

The level of lamin A at the NE is also regulated by phosphorylation that facilitates the shuttling of lamin A between the lamina and nucleoplasm. Therefore, the identification of the relevant kinases and phosphatases would represent an important step in understanding the process. While several kinases have been already identified for the specific phosphosites, the counteracting phosphatases are still elusive. Despite the importance of lamin A biology, not a single protein phosphatase has been so far identified.

Previous work reported that the de-phosphorylation of this residue requires a SIM in lamin A, a transit onto the anaphase/telophase chromosomes and SUMO2 activity [9].

We have also previously shown that Repo-Man, a PP1 interacting protein that is responsible for recruiting PP1 to the anaphase chromatin, was causing nuclear morphology defects when depleted by RNAi in HeLa cells [10]. Repo-Man has a SUMOylation site that, when mutated, causes a decreased de-phosphorylation of S22 in lamin A. This study reveals the first identified phosphatase that directly or indirectly, regulates lamin A dynamics. Although the de-phosphorylation could in theory be mediated by a secondary and yet unknown phosphatase, the requirement for Repo-Man SUMOylation and direct binding to lamin A clearly points towards a more direct role in this phospho-switch.

### 4.1. Opening up

These results are quite interesting in many ways. In terms of cancer progression, Repo-Man is overexpressed in many aggressive and late-stage cancers where the high expression level also correlates with a poor prognosis [29]. In this respect, it can be envisaged that, besides the known role in mitosis and chromosome segregation, Repo-Man would maintain low lamin A S22ph levels that, in turn, would provide a thicker lamina at the NE (remember that only the phosphorylated form is soluble in the nucleoplasm), thus acting as a pro-survival feature during the metastatic invasion.

Interestingly, Repo-Man is not expressed (or at a very low level) in differentiated tissues apart from testes and cardiomyocytes (https://www.proteinatlas.org). While for testes it is understandable that the process could be linked to cell division regulation, for the heart, an organ that in humans does not undergo regeneration, it could be linked to the regulation of correct lamina stiffness at the NE. Although further studies will be required to prove a role in this organ, it is worth noting that a heterozygous S22 L mutation in lamin A has been reported in a patient with dilated cardiomyopathy and premature ventricular beats requiring a heart transplant [30]. In future studies, it will be important to relate these phenotypes to the level of S22 phosphorylation, lamin A stiffness and gene expression.

The results we have obtained in this paper also provide an interesting addition to the biology of protein phosphatases. To the best of our knowledge, this represents the first reported possible substrate interaction mediated by SUMO. Our data show that the current idea that Repo-Man/PP1 phosphatase substrate selection is only guided by localization is not correct. Possibly in some cases, such as for lamin A, the interaction with the substrate needs to be further stabilized by a SUMO/SIM interaction. We cannot speculate at this point on why the interaction is transient unless we hypothesize that Repo-Man SUMOylation is very transient too (as the new published paper seems to suggest [24]), thus allowing continual release of the substrate. Once de-phosphorylated, lamin A can start assembling and will be no longer available for binding. This study highlights also the importance of Repo-Man localization. In fact, removing the active complex from the anaphase chromatin has a similar effect to the depletion of the complex. The fact that lamin A S22ph is increased on chromatin, suggests that lamin A is recruited to chromatin independently from Repo-Man by a yet unknown chromosome-associated protein/complex.

These data could imply that the Repo-Man SUMOylation is carried out by a chromosome bound pool of a SUMO modifier. This is consistent with the fact that several SUMO2/3 modified proteins localize to the chromosome arms during anaphase/telophase in human cells [31] and *Caenorhabditis elegans* [32]. In the future, it would be interesting to identify all the Repo-Man substrates in anaphase and understand how common this substrate recruitment strategy is for this complex.

## Data Availability

The microscopy data are available from the corresponding author upon request and will be released via Figshare.
